# The disease burden of respiratory syncytial virus in Infants

**DOI:** 10.1097/QCO.0000000000000952

**Published:** 2023-08-23

**Authors:** Alasdair P.S. Munro, Federico Martinón-Torres, Simon B. Drysdale, Saul N. Faust

**Affiliations:** a NIHR Southampton Clinical Research Facility and Biomedical Research Centre, University Hospital Southampton NHS Foundation Trust; b Faculty of Medicine and Institute for Life Sciences, University of Southampton, Southampton, UK; c Translational Paediatrics and Infectious Diseases, Hospital Clínico Universitario and Universidad de Santiago de Compostela; d Genetics, Vaccines and Paediatric Infectious Diseases Research Group (GENVIP), Instituto de Investigación Sanitaria de Santiago and Universidad de Santiago de Compostela (USC), Galicia; e CIBER Enfermedades Respiratorias (CIBERES), Instituto de Salud Carlos III, Madrid, Spain; f Centre for Neonatal and Paediatric Infection, Institute for Infection and Immunity, St George's, University of London; g Department of Paediatrics, St George's University Hospital NHS Foundation Trust, London, UK

**Keywords:** bronchiolitis, monoclonal antibodies, respiratory syncytial virus

## Abstract

**Purpose of review:**

To describe the current global burden of respiratory syncytial virus (RSV) in infants and its implications for morbidity, health resources and economic costs.

**Recent findings:**

New prophylactic therapies are on the horizon for RSV in the form of long-acting monoclonal antibodies suitable for healthy infants and maternal immunizations.

**Summary:**

Despite being responsible for significant global infant morbidity and mortality, until recently there have been no effective therapeutics available for healthy infants to protect them from RSV. Several new drugs are likely to be available within the next few years which could help relieve a huge burden on healthcare systems over the coming winters.

## INTRODUCTION

Respiratory syncytial virus (RSV) is a common respiratory pathogen responsible for a large burden of severe respiratory illness in young children, the elderly and immunocompromised. RSV is the most common cause of hospitalization for infants. This article will explore the global burden of disease caused by RSV in infants. 

**Box 1:**
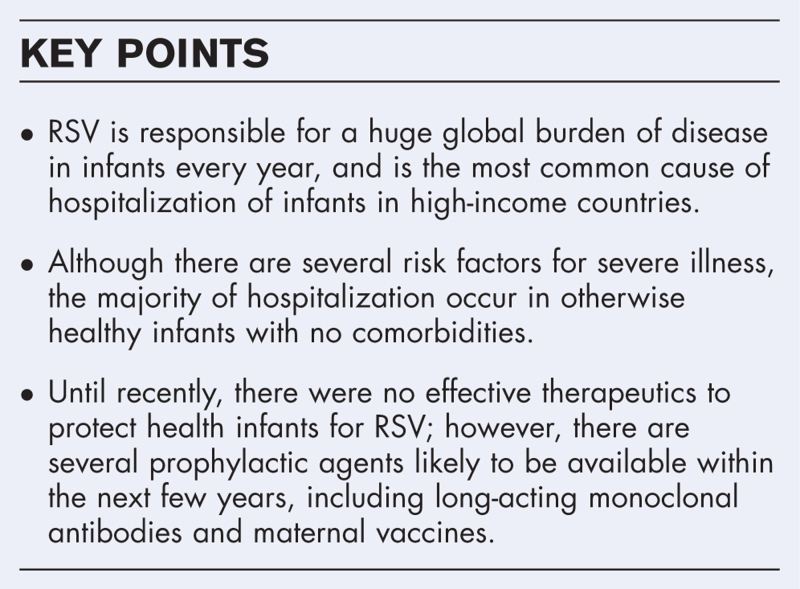
no caption available

## VIRAL STRUCTURE

RSV is an orthopneumovirus from the family *Pneumoviridae*. It is an enveloped, single stranded RNA virus, which is nonsegmented, meaning that it does not undergo the same antigenic shifts responsible for large pandemics that occur with influenza [1]. The virus has two important surface glycoproteins. The G (attachment) protein enables RSV to bind to host cells via glycosaminoglycans, and the F (fusion) protein allows the fusion of the host and viral membranes [2]. The prefusion state of the F protein contains a major antigenic site, which serves as a target for neutralizing antibodies [3]. RSV can be divided into A and B antigenic subtypes depending on the reactivity of the G surface protein to monoclonal antibodies. The A subtype is considered to be more virulent and usually more prevalent, although both circulate simultaneously [4].

## TRANSMISSION AND SEASONALITY

Infants infected with RSV produce very high amounts of infectious virus in respiratory secretions for up to or over 1 week [5], and the virus survives extremely well on hard surfaces and fomites for over 5 h [6]. Studies of transmission within hospital environments found a dramatic reduction in nosocomial infections where staff adhered to strict use of glove and gown protective equipment [7]. Studies utilizing different modes of exposure to nurse volunteers found that direct contact with infants or surrounding surfaces was associated with strikingly high rates of infection as compared with those who were merely sat near to the infants for extended durations [8]. Infants infected with RSV can produce aerosolized particles capable of reaching the small airways, suggesting the potential for airborne transmission [9].

Regional outbreaks of RSV have historically occurred in regular, predictable patterns over winter in temperate climates [10^▪^]. The causes behind these regular patterns are complex but are thought to be driven by both changes in population immunity because of birth rate and waning existing immunity and perhaps to seasonal changes in weather and the environment such as humidity [11]. The impact of background population immunity has come to the fore following the COVID-19 pandemic, after which many regions experienced unseasonal resurgences of RSV with peaks of their epidemic waves over three times higher than would normally be experienced [12^▪^].

## PATHOGENESIS

RSV infects airway epithelial cells, and after replicating for several days infected cells slough into the smaller bronchioles of the lower airway causing obstruction [13]. Although RSV appears to be significantly less cytopathic *in vitro* than viruses such as influenza [14], it is thought that the sloughing of ciliated columnar cells from the upper respiratory tract is what facilitates transmission into the lower respiratory tract [15]. The subsequent cellular debris occluding the bronchioles is not only formed from mucus, cell debris and DNA [16] but also appears to consist of a large degree of neutrophil infiltration [17]. This is accompanied by oedema of the submucosa and adventitial tissue leading to further obstruction of the small airways [16].

Bronchiolitis, the most frequent presentation of RSV infection in infants caused by obstruction of the bronchioles, usually presents with initial symptoms of upper respiratory infection, leading over several days to persistent cough, increase rate and work of breathing, often with auscultatory findings of crackles and wheeze. Clinical examination findings can vary greatly even over short periods of time, as airway debris accumulates or is cleared by coughing or movement of the child [18].

## RISK FACTORS FOR DISEASE

The greatest risk factor for childhood RSV disease is young age, with infants having the highest risk below the age of 3 months and the risk gradually decreasing thereafter [19]. Other risk factors for severe illness include male sex, prematurity, existing lung disease or congenital heart disease [20]. The presence of older siblings in the home also increases the risk of RSV hospitalization [21]. Importantly for both population disease burden and overall healthcare costs, the major of burden of RSV disease occurs in children without existing comorbidities [22]. Whilst there is a substantial body of literature on the impact of comorbidities, and particularly prematurity, on the severity of RSV infection, the majority of hospitalizations occur in children who are previously healthy. Estimates vary between settings, with data from South Africa finding 50% of infants to be previously healthy [23], up to 70% in Switzerland [24], 90% in Israel [25] and 95% in Northern Spain [22].

## BURDEN OF DISEASE

RSV is one of the most important pathogens of early childhood, thought to infect almost all children before the age of 2 years [26]. RSV is responsible for substantial morbidity and mortality worldwide. A global review of RSV burden estimated that in 2019, there were 33 million RSV-associated lower respiratory infection episodes in young children, with 95% of these occurring in low-income and middle-income countries [27^▪▪^].

## HOSPITALIZATION

In high-income countries, RSV is the largest cause of hospitalization in children. The majority of this burden falls on otherwise healthy children during the first year of life [27^▪▪^,^28^,^29^]. In the UK, RSV-attributable disease is estimated to result in between 20 000 and 30 000 hospitalizations every year, significantly higher than for influenza (up to 20 times higher for children under 6 months) and accounts for over 55 000 annual bed days [30,31]. In France, RSV causes an average of 45 000 hospitalizations per year, 69% of which are in infants less than 1-year old, representing 28% of all hospitalizations in this age group [32]. Studies in the United States, utilizing data from the healthcare cost and utilization project found the annual rates of hospitalization for children less than 1-year old to be 2381 per 100 000 children, compared with 181 per 100 000 for influenza [29]. However, a systematic review has highlighted variations in the annual incidence of infant hospitalization depending on the methodology used to measure and calculate the rate; active surveillance studies (11 per 1000) compared with administrative claims (21 per 1000) and modelling approaches (23 per 1000) [33]. The pooled incidence in the United States was 19.4 per 1000 [33]. In South Africa, a study using private hospital administrative data found the all-respiratory hospitalization rate from RSV among children less than 1-year old was 7601 per 100 000 person-years [34]. A systematic analysis estimated that worldwide, RSV accounts for around 3.6 million lower respiratory tract hospitalizations in young children per year, with 1.4 million of these occurring in children 0–6 months old [27^▪▪^]. Across Europe, the average length of stay for RSV respiratory infections ranges from 2 to 4 days, and RSV is thought to account for between 9.9 and 21.2 hospital bed days per 1000 children less than 5 years old annually [35]. In addition, a year-on-year increase in the number of hospitalizations because of RSV since 2004 has been noted in a variety of settings and studies [36].

**FIGURE 1:**
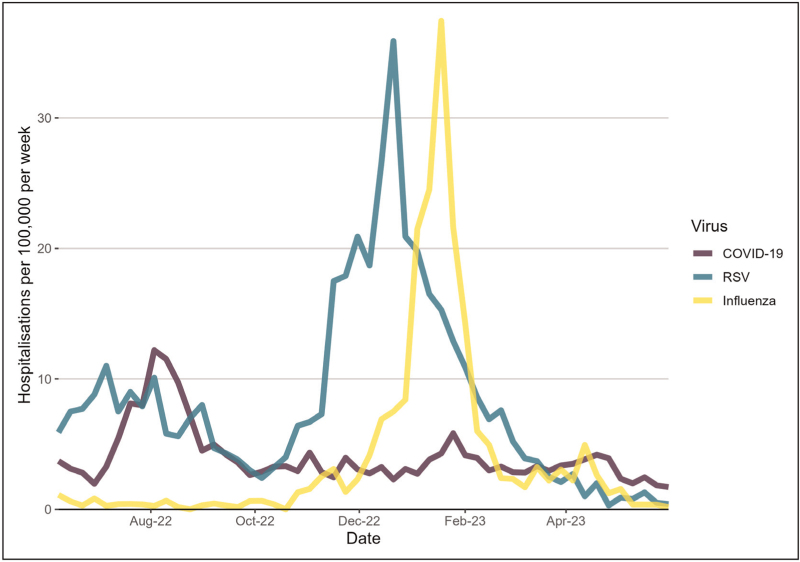
Respiratory virus hospitalizations in under 5's in England. Rates of weekly hospitalizations of less than 5-year-olds per 100 000 population by SARI Watch, England, of COVID-19, RSV and influenza during winter of 2022/2023 [37]. RSV, respiratory syncytial virus.

The COVID-19 pandemic puts RSV hospitalization figures into sharp focus. Data from the UK Health Security Agency SARI Watch surveillance system, allows us to compare the rates of hospitalizations of children under 5 years of age for RSV, influenza and COVID-19 during the winter of 2022/2023 when all three were in circulation together for the first time [37] (Fig. 1).

## OUTPATIENT BURDEN OF DISEASE

Whilst RSV is well recognized for its impact on hospitalization of young children, it is also an important cause of morbidity in outpatient and primary care settings. A prospective cohort study of 5067 children from the USA found that RSV was associated with 15% of all paediatric office visits for respiratory infections between November and April, with rates of visits for children under 5 years old three times higher than to emergency departments [38]. A separate prospective study of 431 newborn infants found a seasonal incidence of RSV illness of 328.4 per 1000, with acute otitis media developing in 76.9% of RSV-infected infants, 70% of whom required antibiotic treatment [39]. Modelling from the UK estimates over 450 000 GP episodes attributable to RSV per year in the UK [30]. A prospective cohort study from Kenya found an average incidence of 22.3 per 1000 persons for medically attended influenza like illness for children under 5 years of age [40]. It is estimated that in the USA, over 700 000 workdays are missed annually by caregivers of children with RSV infections, well over double that of which are missed because of influenza [41]. Whilst the majority of these children will have no underlying health problems, the incidence amongst children with risk factors such as chronic lung disease may be as high as 272 per 1000 children [42]. A systematic review suggested that the true burden of RSV on healthcare resources in outpatient setting has not been fully recognized [43].

## MORTALITY

Whilst the majority of children with severe RSV infection will recover, RSV is responsible for a considerable burden of early childhood mortality. Recent estimates show that RSV is responsible for over 100 000 deaths annually in children less than 5 years old worldwide. Over 45 000 deaths are in children 0–6 months old, accounting for 3.6% of all deaths in children 28 days to 6 months old [27^▪▪^]. Strikingly, 97% of these deaths are estimated to occur in low-income and middle-income countries [27^▪▪^]. The case fatality rate of RSV differs significantly depending on the presence of risk factors, with rates for otherwise healthy children being consistently less than 1% [44]. Factors such as nosocomial infection, prematurity, congenital heart disease, trisomy 21, low birth weight and HIV infection increase the risk of mortality, although estimates of the effect size vary [44–46]. A substantial decrease in in-hospital mortality has been observed over time among developing countries (0.99% prior to 2012 and 0.54% since 2012), although a much more limited decrease has been observed in industrialized countries (0.11 vs. 0.08%) [27^▪▪^].

## ECONOMIC IMPACT

The large illness and treatment burden of RSV is also associated with a huge social and economic cost. The annual hospitalization cost in France increased from €93.2 million in 2010 to €124.1 million in 2017, with 80% of the economic burden represented by infants less than 1 year old [32]. A systematic review in the United States estimated annual mean inpatient costs per RSV patient to be between $9825 for full-term infants to $26 120 for preterm infants [47]. Despite increased costs per premature infant, full-term infants are responsible for 80% of hospitalizations and 70% of the costs of RSV hospitalizations in the United States, with RSV treatment costs totalling $709.6 million annually [48]. The RAND Corporation estimates that RSV in children under 5 years old in the UK is responsible for £14 million in lost productivity of parents and carers, £1.5 million in out-of-pocket costs, and £65 million in healthcare costs, adding up more than £80 million each year [49]. Nearly 50% of the annual RSV costs are incurred by children less than 1 year old, and 19% of the costs are attributable to infants born prematurely [49]. Statistical modelling estimates that the global economic burden comes to a total treatment cost of $611 million in indirect costs, and 1.2 million disability-adjusted life years [50]. In 2017, the global cost of medical management of RSV infections in young children was estimated to be €4.82 billion (95% confidence interval (CI) 3.47–7.93), with 55% of the costs accounted for by hospitalizations and 65% of the total accrued in developing countries [51].

## THERAPIES

Multiple vaccines are currently in development, with phase 3 results of a bivalent prefusion F vaccine during pregnancy achieving 81.8% efficacy at preventing severe lower respiratory tract illness [52^▪▪^]. The only licenced prophylactic agents are monoclonal antibodies (mAb). Palivizumab is in current use, administered by monthly injection during the RSV season, and only available for the highest risk infants. The newly approved mAb nirsevimab [53], which can be given as a single dose covering a whole RSV season [54] has been shown in a meta-analysis of phase 2 and 3 trials to reduce medically attended RSV lower respiratory infection by 79.5% (95% CI 65.9–87.7%) [55^▪▪^]. More recently, the interim results of a large, phase 3 clinical trial in Europe of nirsevimab are more representative of ‘real world’ settings, demonstrated an 83% (95% CI 67.6–92.04) reduction in RSV hospitalization in infants under 12 months of age, and a 58% (95% CI 39.69–71.19) reduction in all-cause lower respiratory tract disease hospitalization [56].

## CONCLUSION

Childhood RSV disease is associated with a substantial global health and economic burden. Whilst risk factors, such as prematurity and congenital heart disease are associated with much greater risk of severe illness, the majority of the costs and disease are associated with infection in children without comorbidities. There are no widely used specific therapies for RSV, and treatment for lower respiratory tract infections are predominantly supportive. This may be a pivotal moment in the history of RSV management, with several effective preventive medicines likely to become available in the next few years. Logistics and parental acceptance and uptake are likely to be important factors in deciding which of these therapies to deploy to populations at large. It is important to ensure equitable distribution of these medicines worldwide, as the majority of severe illness occurs in low-income and middle-income countries.

## Acknowledgements

*None.*

### Financial support and sponsorship

*A.P.S.M. and S.F. both have their salary in part funded by the NIHR Southampton Clinical Research Facility and Biomedical Research Centre. F.M.-T. has received support for the present work from the Instituto de Salud Carlos III (Proyecto de Investigación en Salud, Acción Estratégica en Salud) ‘Fondo de Investigación Sanitaria’ (FIS; PI070069/PI1000540/PI1601569/PI1901090) from “plan nacional de I+D+I”.*

### Conflicts of interest

*S.N.F. acts on behalf of University Hospital Southampton NHS Foundation Trust as an Investigator and/or providing consultative advice on clinical trials and studies for paediatric and adult vaccines and antimicrobial agents funded or sponsored by manufacturers including Sanofi (including for the HARMONIE trial and RSV vaccines), Janssen, Pfizer, Moderna, AstraZeneca, GlaxoSmithKline, Novavax, Seqirus, Medimmune, Merck and Valneva. He receives no personal financial payment for this work. Federico Martinón-Torres has received honoraria from GSK group of companies, Pfizer Inc, Sanofi, MSD, Seqirus, Biofabri, and Janssen for taking part in advisory boards and expert meetings and for acting as a speaker in congresses outside the scope of the submitted work. F.M.-T. has also acted as principal investigator in randomized controlled trials of the above-mentioned companies, as well as Ablynx, Gilead, Regeneron, Roche, Abbott, Novavax, and MedImmune, with honoraria paid to his institution. S.B.D. has received honoraria from MSD and Sanofi for taking part in RSV advisory boards and has provided consultancy and/or investigator roles in relation to product development for Janssen, AstraZeneca, Pfizer, Moderna, Valneva, MSD, iLiAD and Sanofi with fees paid to St George's, University of London. S.B.D. is a member of the UK Department of Health and Social Care's (DHSC) Joint Committee on Vaccination and Immunisation (JCVI) RSV subcommittee and Medicines and Healthcare products Regulatory Agency's (MHRA) Paediatric Medicine Expert Advisory Group (PMEAG), but the reviews expressed herein do not necessarily represent those of DHSC, JCVI, MHRA or PMEAG.*

## REFERENCES AND RECOMMENDED READING

Papers of particular interest, published within the annual period of review, have been highlighted as:

▪ of special interest
▪▪ of outstanding interest
